# A Remote Chronic Disease Management Program to Improve Cardiovascular and Metabolic Outcomes in a Diverse Community‐Based Diabetic Population: Study Design of the TAMIS Trial

**DOI:** 10.1111/jep.70456

**Published:** 2026-04-29

**Authors:** Renato de Carvalho Barros, Andrea Stephanus, José Antonio Barbosa Filho, Evellyn Mariana, Yasmim Botelho, Thaiene M. M. Severino, Robson Conceição Silva, Lucila de Jesus Almeida, Cristiane Koeche, Bruno Gedeon, Mariana Guimarães Souza de Oliveira, Ana Carolina Augusto, Catarina Ferraz, F. Enzo, Gabriela de Lima, Julia Andrade Ibiapina, Giselle Pinto, Sérgio Henrique Rodolpho Ramalho, Ana Claudia C. Nogueira, Andrei C. Sposito, Alessandra M. Campos‐Staffico, Alexandre Anderson S. M. Soares, Luiz Sérgio F. de Carvalho

**Affiliations:** ^1^ Data Laboratory for Research on Quality of Care and Outcomes (LaDaQCOR) Catholic University of Brasília (UCB) Brasília Brazil; ^2^ Post‐Graduation Program in Medical Sciences University of Brasília Brasília Distrito Federal Brazil; ^3^ Research Group on Cardiovascular Diseases of Brasília, Higher School of Health Sciences (ESCS) University of the Federal District (UnDF) Brasília Brazil; ^4^ Postgraduate Program in Physical Education (PPGEF) Catholic University of Brasília (UCB) Brasília Brazil; ^5^ Hospital Sírio Libanês Brasília Distrito Federal Brazil; ^6^ Aramari Apo Institute Brasília Brazil; ^7^ Department of Cardiology State University of Campinas (Unicamp) Campinas (SP) Brazil; ^8^ Department of Pharmaceutical Sciences, School of Pharmacy and Health Professions Creighton University Omaha USA; ^9^ Clarity Healthcare Intelligence Campinas (SP) Brazil

**Keywords:** cardiovascular diseases, predictive models, public health, telemedicine

## Abstract

**Introduction:**

Cardiovascular and cerebrovascular diseases are projected to expand in the coming decades as the main cause of death. This study outlines the escalating burden of cardiovascular diseases in low‐ and middle‐income countries (LMICs), particularly in Latin America (LA), which is expected to surpass that of other high‐population regions. Despite advances in prevention strategies, adherence to therapeutic goals remains a global challenge, especially in LMICs where control rates are notably low for conditions such as hypertension, type 2 diabetes mellitus (DM2), and dyslipidemia. Emerging evidence from LMICs supports the feasibility and effectiveness of telehealth interventions for cardiometabolic disease management in primary care.

**Aims:**

To address these challenges, a remote chronic disease management program has been developed to optimize risk factor control through standardized medical protocols and task automation.

**Methods:**

This is an open‐label randomized and decentralized clinical trial describing the design and methodology of the TAMIS program, which will assess its impact on community‐dwelling diabetic subjects with uncontrolled HBA1c and uncontrolled LDL‐C and/or blood pressure. Individuals will be randomized to usual care or remote care and followed for 84 weeks. Remote monitoring offers a practical response to the limited capacity of LMIC health systems to ensure sustained follow‐up and continuity of care for chronic conditions. The primary endpoint is a win‐ratio of hierarchical events, including all‐cause deaths and the frequency of all‐cause hospitalizations. The secondary endpoints include control of HbA1c, LDL‐C and blood pressure according to clinical guidelines, as well as patient‐reported outcomes and clinical parameters, including blood pressure, glycemic control, and LDL cholesterol levels. Subgroup analyses will explore the impact of demographics and clinical characteristics on treatment outcomes. Overall, the study aims to improve health care delivery, enhance patient education, and foster collaboration between patients and health care providers to mitigate the growing burden of cardiovascular diseases in LMICs.

## 1 Introduction

Cardiovascular and cerebrovascular diseases are projected to become the leading cause of death in low and middle‐income countries (LMICs) by 2040 [1]. Among these nations, Latin America (LA) is expected to surpass both China and India, exhibiting the highest rate of cardiovascular disease‐related deaths per inhabitant [1]. Currently, ischemic heart and cerebrovascular conditions account for nearly 30% of global mortality and an alarming 69% of deaths among individuals aged 20−59 years in Brazil [2, 3]. This trend is particularly concerning as it disproportionately affects individuals in their most productive years, leading to increased morbidity, economic burden, and loss of workforce productivity in LA's adult population [4]. While developed countries have seen a decline in ischemic disease incidence, LMICs continue to struggle with limited health care access, suboptimal preventive strategies, and treatment gaps [5, 6]. Non‐communicable diseases (NCDs) now represent the predominant health challenge in these settings, generating an annual economic impact exceeding 1.2 trillion US dollars [7].

Despite advancements in prevention and treatment, adherence to therapeutic goals remains a major challenge worldwide [8]. Even in high‐income countries such as Canada and Sweden, only about 50% of individuals with type 2 diabetes (DM2), systemic arterial hypertension (SAH), or dyslipidemia achieve proper disease control. In contrast, hypertension control rates in LA remain critically low, ranging from 5% to 8% [8]. In Brazil, these figures are equally concerning, with only 30.6% of individuals achieving target SBP, 18.8% reaching LDL‐C goals, and 41% controlling for HbA1c levels [9].

Traditional interventions such as continuing medical education, patient awareness campaigns, and financial incentives have led to a decline in acute cardiovascular events in developed countries. However, LMICs still face significant obstacles in early diagnosis, treatment accessibility, and long‐term disease management [5, 10].

To bridge these gaps, we have developed the TriAl on the remote Management and control of blood pressure, glycemia, and lipids in a diverse community‐baSed population—Artificial Intelligence platform (TAMIS‐IA), a structured and remotely delivered chronic disease management program. TAMIS‐IA integrates guideline‐based, algorithm‐driven clinical workflows for hypertension, diabetes, and dyslipidemia management. For hypertension, patients perform structured home blood pressure monitoring during 6 consecutive days, with automated calculation of aggregated blood pressure averages guiding predefined titration pathways. For diabetes, remote follow‐up includes structured lifestyle assessment, pharmacologic adjustment according to algorithms based on the American Diabetes Association (ADA) guidelines, and scheduled laboratory reassessments. LDL‐C management follows a stratified risk‐based protocol incorporating laboratory safety parameters and contraindication screening prior to therapeutic escalation. All therapeutic recommendations generated by the system are reviewed and approved by supervising physicians before implementation.

This study will evaluate the clinical impact of TAMIS‐IA on major clinical outcomes, providing valuable evidence on the effectiveness of remote chronic disease management models in LMICs. By leveraging technology‐driven interventions, this trial aims to establish a scalable, cost‐effective, and sustainable strategy for improving long‐term cardiovascular risk management in underserved populations.

## 2 Methods

### 2.1 Trial Design

**Figure 1:**
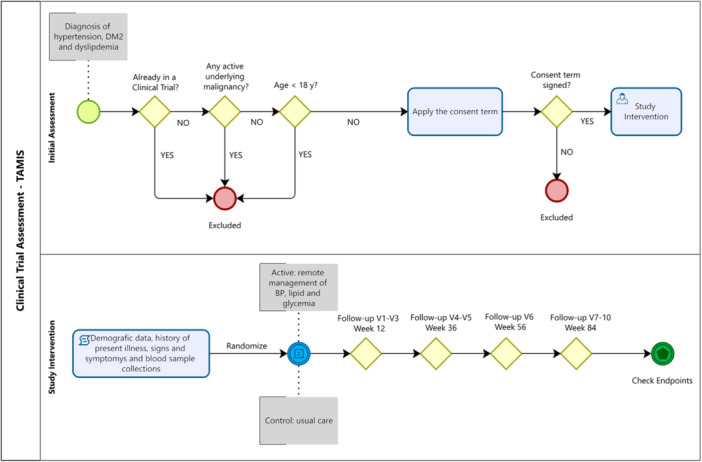
Overview of patient flow and study design.

This prospective, multi‐center clinical trial aims to evaluate the impact of a remote management program for lipid levels, glucose, and hypertension in Brazilian adults. Eligible participants who meet the inclusion and exclusion criteria will be followed for 84 weeks, with predefined endpoints assessed at specific time points. The flow of patient selection, randomization, and intervention processes is summarized in Figure 1.

Participant selection and randomization involve confirming interest, explaining the study details, and assessing eligibility. Baseline demographic data, medical history, and health status will be collected before randomization. During follow‐up visits, the study team will: (i) measure blood pressure and glycemic levels; (ii) administer the study protocol; (iii) monitor symptoms and adverse events, and (iv) evaluate treatment adherence.

Adherence will be defined as the completion of at least 80% of scheduled home measurements and the use of at least 80% of prescribed medication doses during follow‐up. Data will be obtained through the TAMIS‐IA platform and scheduled study visits. Participants meeting both criteria will be classified as adherent in secondary analyses. Low adherence will be addressed through sensitivity analyses.

**Figure 2:**
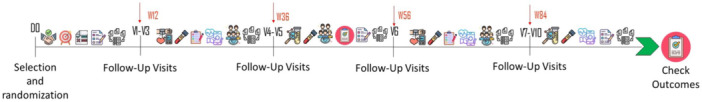
Study Schedule.

At baseline and weeks 12, 24, 36, 56, and 84, blood samples will be collected, laboratory tests performed, and study endpoints assessed. At week 84, similar follow‐up procedures will take place, with additional endpoint evaluations and final blood sample collection. In addition to these scheduled in‐person laboratory assessments, follow‐up incorporates structured remote contacts and digital data capture. Participants are not required to attend in‐person visits for each follow‐up interaction. In the intervention arm, counseling and medication titration discussions are conducted remotely by trained monitors under physician supervision. Throughout the study, all data will be systematically documented and entered into a secure database. Additionally, quality of life will be assessed using the European Quality of Life 5 Dimensions (EQ‐5D) and the ICEpop Capability Measure for Adults (ICECAP‐A) at multiple time points. The description of the study procedures and measurements is provided in Supporting Information Material 1: Schedule of Activities and illustrated in Figure 2.

This trial has been submitted to the Brazilian Registry of Clinical Trials (ReBEC) and registered as a clinical trial under number #15832.

## 3 Participants

This will be a multi‐center study enrolling adult patients (≥ 18 years old), regardless of sex, from 20 Basic Health care Units (BHUs) from the Federal District (DF, Brazil). Participants will be randomly selected from the pool of registered diabetic individuals meeting trial eligibility.

### 3.1 Eligibility Criteria

- Inclusion criteria
  i. Adults aged ≥ 18 years, receiving care within the Brazilian Primary Health Care Network.
  ii. At least one documented consultation with a primary care physician (PCP) within the last 36 months.
  iii. Ability to provide informed consent personally or via a legally authorized representative.
  iv. Access to a telephone or digital platform for remote follow‐up.
  v. Diagnosis of type 2 diabetes mellitus with glycated hemoglobin (HbA1c) ≥ 7.0% measured in a certified laboratory within the previous 18 months.
  vi. Presence of at least one additional uncontrolled cardiovascular risk factor at baseline:
    − Uncontrolled hypertension, and/or
    − Uncontrolled LDL‐C.

#### 3.1.1 Operational Definitions

− Uncontrolled hypertension: mean 24‐h ambulatory blood pressure ≥ 130/80 mmHg, or ≥ 2 office or home BP readings ≥ 130 mmHg systolic and/or ≥ 80 mmHg diastolic recorded in the electronic health record (EHR) within the past 18 months, excluding emergency or procedural visits.
− Uncontrolled LDL‐C: (i) LDL‐C ≥ 50 mg/dL in individuals at very high cardiovascular risk; (ii) LDL‐C ≥ 70 mg/dL in individuals at high cardiovascular risk; (iii) LDL‐C ≥ 100 mg/dL in individuals at intermediate cardiovascular risk; (iv) individuals with a history of LDL‐C ≥ 190 mg/dL at any time, according to the 2025 Brazilian Guideline on Dyslipidemias and Atherosclerosis Prevention. Individuals with diabetes mellitus may present cardiovascular risk ranging from intermediate to very high, depending on disease duration, the presence of additional risk factors, or evidence of target‐organ damage.

- Exclusion criteria
  i. Secondary hypertension or conditions requiring non‐standard management.
  ii. Participation in another interventional clinical trial.
  iii. Individuals with active underlying malignancies.
  iv. Moderate‐to‐severe cognitive impairment or severe psychiatric condition that precludes consent or adherence.
  v. Inability to use or access a telephone for remote contacts, even with caregiver assistance.
  vi. Illiteracy that prevents comprehension of informed consent or digital interaction.

The exclusion of illiterate individuals was defined to ensure valid informed consent and reliable participation in remote data collection and digital self‐monitoring. Recruitment occurs in community‐based primary care services serving socioeconomically diverse populations, and results will be interpreted in the context of individuals with functional literacy compatible with digital self‐care within the public health system.

## 4 Ethical Considerations and Good Clinical Practice (GCP) Compliance

The study protocol was reviewed and approved by the Research Ethics Committee of the Foundation for Education and Research in Health Sciences (FEPECS), under opinion number 7.348.902, and registered in the national system with the Certificate of Ethical Appreciation Submission (CAAE) number 74170623.0.0000.5553. All participants will provide written informed consent prior to enrollment, in accordance with Brazilian Resolution CNS 466/2012 and international GCP guidelines.

## 5 Interventions

Participants in the control arm will receive treatment at the discretion of their physician, without interference from the study personnel, and they will be followed up through app interactions, phone calls, and lab exams every 24 weeks.

Both study arms are recruited and followed within the same BHU of the Brazilian Primary Health Care Network, ensuring comparable clinical settings and multidisciplinary staff. Usual care in these units is provided by PCPs and nurses and is defined as routine management typically involving in‐person consultations every 3–6 months according to clinical need, with medication adjustments and laboratory testing performed at the physician's discretion, and no structured digital follow‐up. In the control arm, no standardized algorithms or automated decision‐support tools are used, and there are no scheduled tele‐touchpoints beyond those required for data collection. All usual‐care contacts and visit frequencies will be documented to allow comparison of exposure intensity between study arms. To minimize contamination between study arms, the structured remote workflow is delivered exclusively by the trained study monitors assigned to the intervention arm. Control participants do not have access to TAMIS‐IA clinical decision support, automated titration protocols, or structured telemonitoring beyond protocol‐required data collection. Usual care providers remain independent from the intervention workflow, and no algorithm‐driven recommendations are integrated into routine care.

Participants in the active intervention arm will receive treatment based on standardized clinical guidelines, with instructions provided remotely by study monitors under physician supervision. The study monitors are health care professionals and research fellows with clinical backgrounds, specifically trained to conduct structured telephone follow‐ups, data collection, and patient education activities. The intervention is supported by TAMIS‐IA, a web‐based digital health platform that enables remote management, patient monitoring, and clinical decision support. All medication adjustments or new prescriptions generated through the digital platform are reviewed and formally approved by physicians before implementation to ensure clinical safety and protocol compliance. Further technical and operational details of the platform are provided in Supporting Information S1: Material 2.

TAMIS‐IA is a non‐commercial digital health platform developed collaboratively by academic and public institutions for research and service improvement. The platform's software and algorithms are maintained under institutional ownership, with no private licensing, profit generation, or commercial partnerships.

All members of the study team involved in follow‐up and data collection underwent structured and standardized training prior to participant contact. The training program included protocol‐based lifestyle counseling focused on weight management, physical activity, sodium reduction, alcohol moderation, and smoking cessation; application of guideline‐directed pharmacologic algorithms for hypertension, diabetes, and dyslipidemia; documentation procedures and data entry within the TAMIS‐IA platform; standardized telephone communication techniques; and identification and reporting of adverse events. Training was delivered by supervising physicians and senior investigators with expertise in cardiovascular and metabolic disease management. Preparatory training sessions were conducted regularly over the year preceding trial initiation through weekly 60‐min meetings focused on protocol familiarization, case discussions, and workflow standardization. Completion of formal training in international GCP, in accordance with International Council for Harmonization (ICH) guidelines, was a prerequisite for participation in study‐related activities. Competency was verified before participant interaction, and ongoing supervision is maintained throughout the trial to ensure protocol adherence and clinical safety.

Data monitoring will be performed remotely by the coordinating center, which maintains a full audit trail of user access, data entry, and edits. Automated consistency checks and range validations are integrated into the electronic data capture platform, and all queries or discrepancies are reviewed and resolved centrally before database lock to ensure data integrity.

Safety assessments are described in Supporting Information Material 3, including the classification, documentation, and reporting of adverse events.

### 5.1 Lifestyle Management

At the beginning of the study, trained study monitors will interview patients to assess their health habits and provide individualized counseling on lifestyle modifications. The educational approach will emphasize weight loss, physical activity, and the reduction of sodium and alcohol consumption. Patients will have continuous access to navigators, who can answer questions and address concerns. During every follow‐up call, lifestyle modifications will be reviewed, with specific counseling on smoking cessation, including access to resources for quitting. Additionally, medication adherence will be reinforced through the recommendation of once‐daily drug regimens and combination therapies. Each call will also include a review of individual treatment goals.

### 5.2 Blood Pressure Management

**Figure 3:**
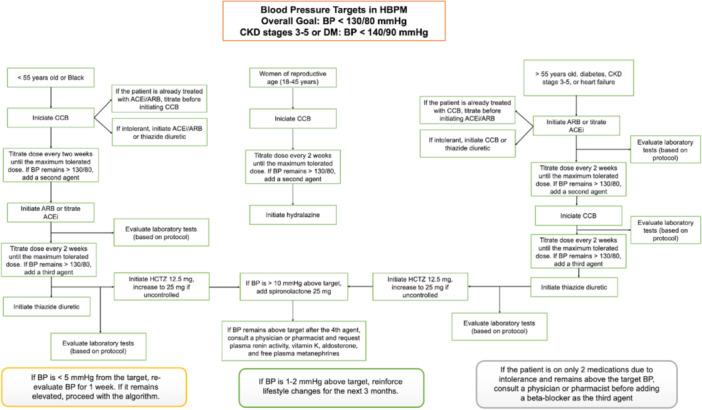
Hypertension management algorithm: treatment initiation, titration, and referral pathways. ACEi, angiotensin‐converting enzyme inhibitor; ARB, angiotensin receptor blocker; BP, blood pressure; CCB, calcium channel blocker; CKD, chronic kidney disease; DM, diabetes mellitus; HBPM, home blood pressure monitoring; HCTZ, hydrochlorothiazide.

A SAH treatment algorithm was developed based on the 2025 Brazilian Hypertension Guideline [11]. Figure 3 illustrates the clinical workflow for SAH management, including the initiation and titration of pharmacological treatment. Upon enrollment, patients will measure their BP at home for six consecutive days, following a standardized protocol: twice in the morning and twice in the evening, always before taking antihypertensive medications. Patients will receive home BP monitors, equipped with technology that enables real‐time transmission of BP readings to the EHR via Bluetooth.

A customized software platform was developed to automatically calculate weekly BP averages, defining target BP as a weekly systolic BP (SBP) < 130 mmHg and diastolic BP (DBP) < 80 mmHg. The system allows for dynamic adjustments of BP targets based on individual clinical indications.

For patients whose home BP remains elevated, medication adjustments will be conducted remotely, via telephone consultations with a patient monitor, following the clinical algorithm integrated into the software platform. Physicians will review and approve all new prescriptions before implementation. This approach allows for more frequent medication titrations than in standard clinical practice.

After each titration, patients will wait 1 week for stabilization before repeating another 6‐day BP measurement cycle at home. This results in a biweekly cycle of medication titrations, which aligns with the pharmacokinetics of the prescribed antihypertensive agents, including angiotensin‐converting enzyme (ACE) inhibitors, angiotensin‐receptor blockers (ARBs), calcium channel blockers (CCBs), and diuretics.

This accelerated titration protocol aims to overcome the therapeutic inertia commonly seen in traditional BP management. Throughout the study, electrolytes and renal function will be monitored when clinically indicated. Once patients achieve BP control, they will transition out of the intensive titration phase, moving to a surveillance phase with follow‐ups every 6 months.

If a participant's home BP remains elevated despite the use of three antihypertensive agents, including a diuretic, they will be classified as having resistant hypertension and automatically referred to a SAH specialist for further evaluation and treatment.

### 5.3 Glucose Management

**Figure 4:**
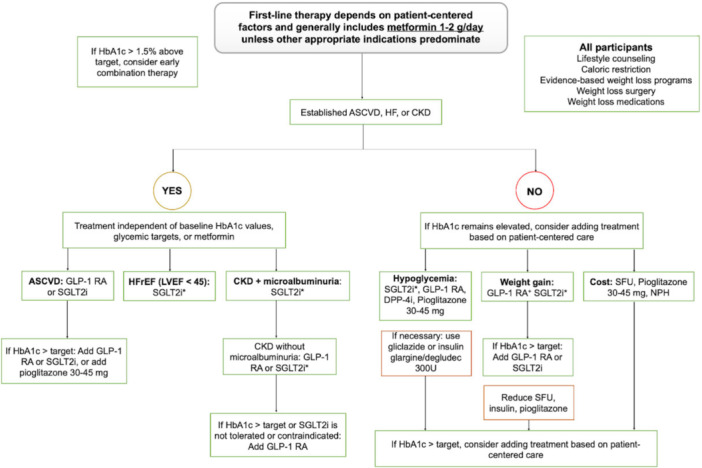
Diabetes management algorithm: stratification, treatment, and monitoring strategies. *If eGFR is adequate. ^Empagliflozin and dapagliflozin have shown benefits in dedicated HF trials. Canagliflozin demonstrated a reduction in HF hospitalization in cardiovascular outcome trials. #Dapagliflozin and canagliflozin showed benefits in kidney outcome studies. Empagliflozin demonstrated a reduction in CKD progression in cardiovascular outcome studies. + Weight loss is greater with semaglutide > liraglutide > dulaglutide > exenatide > lixisenatide. ASCVD, atherosclerotic cardiovascular disease; CKD, chronic kidney disease; DPP‐4i, dipeptidyl peptidase‐4 inhibitor; eGFR, estimated glomerular filtration rate; GLP‐1 RA, glucagon‐like peptide‐1 receptor agonist; HF, heart failure; HFrEF, HF with reduced ejection fraction; SFU, sulfonylurea; SGLT2i, sodium‐glucose cotransporter‐2 inhibitor; TZD, thiazolidinedione.

A pharmacologic treatment algorithm for hyperglycemia and diabetes was developed based on the ADA guidelines [12]. Figure 4 illustrates the clinical workflow for diabetes management, detailing the stratification of patients according to cardiovascular risk, comorbidities, and therapeutic goals. First‐line therapy will primarily involve metformin, unless other compelling indications warrant a different approach. The choice of therapy will be patient‐centered, taking into account clinical history, comorbidities, and individual preferences.

Patients diagnosed with diabetes will be monitored every 4−6 weeks by the study team to assess their adherence to lifestyle modifications and pharmacological treatment. Laboratory tests, including HbA1c, fasting glucose, and capillary blood glucose (or continuous glucose monitoring data), will be reviewed every 12 weeks. During the scheduled visit week in which laboratory testing is planned, participants will be instructed to undergo blood sample collection at the designated research laboratory affiliated with the study. Laboratory results are securely shared with patients and monitors, and trained staff record verified values in the study database. Additionally, study monitors will recommend visits to the PCP every 12–24 weeks to screen for signs of neuropathy or polyneuropathy.

All prescriptions will follow a structured protocol, supervised by experienced physicians. Medication doses will be adjusted as needed to ensure patients achieve their glycemic targets, such as HbA1c levels, considering their cardiovascular and renal status. Therapy adjustments will also account for the risk of hypoglycemia, cost‐effectiveness, and patient preferences.

### 5.4 LDL‐C Management

**Figure 5:**
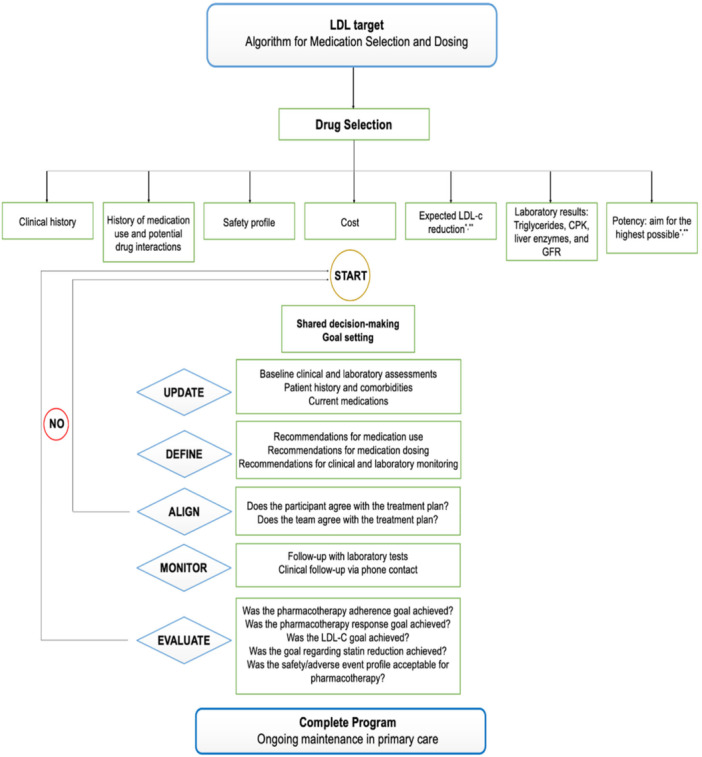
Algorithm for LDL‐C management: medication selection, dosing, and monitoring. *Intermediate Risk (Target LDL‐C: < 100 mg/dL): Initiate simvastatin 40 mg/day and titrate the dose based on LDL‐C levels. If necessary, consider adding ezetimibe 10 mg/day or increasing to atorvastatin 20 mg or rosuvastatin 10 mg. **High Risk (Target LDL‐C: < 70 mg/dL) or Very High Risk (Target LDL‐C: < 50 mg/dL): Initiate atorvastatin 40−80 mg/day or rosuvastatin 20−40 mg/day. CPK, creatine phosphokinase; GFR: Glomerular Filtration Rate; LDL‐C, Low‐Density Lipoprotein Cholesterol.

A treatment algorithm for LDL‐C management was developed based on the 2025 Brazilian Guideline on Dyslipidemias and Atherosclerosis Prevention [13]. Figure 5 outlines the clinical workflow for managing LDL‐C, including medication selection, dosing adjustments, and monitoring strategies. The treatment recommendations will include specific regimens for statins, ezetimibe, and strategies for managing statin intolerance. Medication selection and dosing will be tailored according to patient history, considering factors such as concomitant medications, prior ASCVD events, previous lipid‐lowering therapies, and laboratory data, including creatine kinase (CK), estimated glomerular filtration rate (eGFR), alanine aminotransferase (ALT), and aspartate aminotransferase (AST).

Before entering the LDL‐C management program, a manual verification of contraindications to lipid‐lowering therapy will be required. Relative contraindications include ALT or AST ≥ 5 times the upper limit of normal, CK ≥ 10 times the upper limit of normal, eGFR ≤ 30 mL/min/1.73 m^2^, or documented allergies to lipid‐lowering therapy. Absolute contraindications include pregnancy, pre‐conception planning, breastfeeding, hemodialysis, New York Heart Association (NYHA) class II‐IV HF, or severe psychiatric conditions. Any cases with a relative or absolute contraindication will be discussed with the supervising pharmacist and/or cardiologist.

A clinical algorithm software application was developed to generate individualized therapeutic recommendations for lipid management and track each participant's progress. This system integrates demographic, laboratory, medication, and medical history data through an application programming interface (API) server, allowing for the automated generation of treatment recommendations and follow‐up laboratory testing schedules. Additionally, the software incorporates a scheduling tool for follow‐up calls, laboratory testing, documentation, and coordination of patient care management, ensuring compliance with Brazilian General Data Protection Laws (LGPD).

## 6 Outcomes

### 6.1 Primary Efficacy Endpoint

The key primary efficacy endpoint is a standard win ratio composed of time to occurrence of all‐cause death and the number of non‐fatal cardiovascular hospitalizations from randomization to Week 84. These events will be identified using multiple verified data sources, including EHRs, discharge summaries, and official death certificates, and will undergo centralized review by the data management team to ensure accuracy and completeness across sites. The objectivity of these outcomes, combined with centralized validation procedures, minimizes the potential for ascertainment bias.

### 6.2 Secondary Efficacy Endpoints

The key secondary efficacy endpoints are time from randomization to the first occurrence of the composite of the following events:

- 84‐week change in Quality‐of‐life assessment by the EQ‐5D and ICECAP‐A.
- 12‐, 56‐ and 84‐week adherence to home BP measurements.
- 12‐, 56‐ and 84‐week prescription and adherence to anti‐hypertensives, lipid‐lowering, and anti‐hyperglycemic therapies.

### 6.3 Tertiary Efficacy Endpoint

The change in the ‘metabolic target risk score’ (MeTaRiSc) from baseline to week 84. Each individual will be assigned the value 1 for each parameter above the targets, and the sum of the 3 parameters will be divided by 3. The MeTaRiSc is this weighted mean that varies from 0 to 1.

i. +1 if more than 10% of home SBP measurements ≥ 130 mmHg.
ii. +1 point if LDL cholesterol levels are above targets defined by the 2013 ACC/AHA cholesterol guidelines;
iii. +1 point if glycated hemoglobin (HbA1c) levels ≥ 7.0%.

### 6.4 Safety Assessments

Safety assessments will include adverse events, clinical laboratory measurements (chemistry, hematology), and vital signs (systolic and diastolic blood pressure). A complete medical, surgical, and family history will be completed at the randomization visit. All laboratory test results must be evaluated by the investigator as to their clinical significance. Any observations at physical examinations or laboratory values considered by the investigator to be clinically significant should be considered an adverse event.

## 7 Sample Size Calculation

Given that a comprehensive multifactorial intervention targeting intensive cardiovascular risk factor control has been associated with significant reductions in cardiovascular mortality (−57%), cardiovascular events (−59%), and hospitalizations due to heart failure (HF) in patients with diabetes [1], this study was designed using a standard win ratio test. The win ratio test incorporates both all‐cause mortality and all‐cause hospitalizations, using component‐wise hazard ratios (HRs) as effect size estimators.

For the sample size estimation, predefined hazard ratios (HR 0.90 for all‐cause death and HR 0.70 for all‐cause hospitalization) were used, targeting 90% statistical power with a two‐sided *α* = 0.05% and 5% expected attrition. Sensitivity analyses explored plausible variations in baseline event rates and component HRs to confirm robustness of the win‐ratio and power assumptions. A total of 838 participants (419 per treatment arm) will provide adequate power under these scenarios. Sample size calculations were performed in R (v1.2) using the WR package, as described by Mao et al. and Yu et al. [14, 15]. Supporting Information Material 4 summarizes the simulation scenarios and corresponding sample size estimates.

## 8 Blinding

This study will be conducted as an open‐label trial. However, certain personnel will remain blinded to the randomization process. Specifically, the study sponsor, the independent Data Safety Monitoring Board (DSMB), study administrators, and personnel at the supporting organizations and vendors will be unaware of the allocation sequence.

All study data will be collected at participating sites and entered into the TAMIS‐IA platform, a secure, web‐based electronic data capture platform. Participants will be de‐identified using unique alphanumeric codes, and all data will be managed in compliance with the Brazilian LGPD. Data accuracy will be ensured through double‐entry verification, real‐time audit trails, and predefined data validation rules. Quality assurance activities will include biweekly remote audits, centralized query resolution, and continuous oversight by the Coordinating Center.

An independent DSMB composed of experts in cardiology, clinical trials, epidemiology, and biostatistics will oversee the study's safety and scientific integrity. The DSMB will remain blinded to treatment allocation and will conduct closed‐session interim reviews every 12 weeks, evaluating unblinded data for cumulative adverse events, recruitment metrics, and overall risk‐benefit profile. Predefined stopping criteria for harm, futility, or overwhelming efficacy will guide DSMB recommendations.

On the other hand, patients, study investigators, monitors, and clinical staff at the Health care Units will be aware of the allocation. To mitigate biases, the dataset will be de‐identified after data lock, ensuring that the study medication and treatment allocation are concealed from data analysts and statisticians.

## 9 Randomization

### 9.1 Randomization Sequence Generation

The randomization sequence will be generated using a computerized random number generator within RStudio, utilizing the *blockrand* package.

### 9.2 Type of Randomization

A stratified randomization approach will be employed to ensure balance between the study arms. Stratification will be based on key demographic and clinical characteristics, including age (< 55 or ≥ 55 years), gender and prior cardiovascular disease. Within each stratum, randomization will follow a permuted block design with block sizes randomly varying between four and eight, ensuring equal representation of each intervention arm within each stratum.

### 9.3 Implementation

Participant enrollment will be conducted by trained personnel at the primary health care units, who will follow eligibility criteria to identify and obtain consent from eligible individuals. These personnel will remain unaware of the allocation sequence, preserving the integrity of the randomization process.

After data blocking for analysis, the study intervention groups will be de‐identified, ensuring blinding for data analysts and maintaining the study's methodological rigor.

### 9.4 Statistical Methods

All continuous variables will be presented as median and interquartile range (IQR), while categorical variables will be reported as absolute (n) and relative frequency (%).

Descriptive statistics (mean ± standard deviation, median and IQRs) will be generated for each treatment group to summarize demographic and baseline characteristics. Between‐groups comparisons will be conducted within the Intention‐to‐Treat (ITT) population to ensure that the randomization process successfully achieved balance between groups. Differences in baseline characteristics between treatment groups will be tested using a chi‐square test for categorical variables and a two‐tailed *t*‐test with treatment as a factor for continuous variables. These preliminary analyses will serve as an initial assessment of the adequacy of randomization.

The primary endpoint analysis will be performed using a win‐ratio test, which combines time to all‐cause death and the frequency of nonfatal hospitalizations. The win ratio test will follow the original methodology described by Pocock et al. [16]. Beyond hierarchical endpoint evaluation, this approach allows for the integration of multiple endpoint types. Treatment differences will be tested at an alpha level of 0.05, with a sequential procedure for controlling type 1 error. Additional covariate adjustments for time‐sensitive analyses will be performed using a Cox proportional hazards model, incorporating the randomization stratification factor. The HR for treatment groups will be reported along with the 95% confidence interval (CI).

The secondary endpoint analysis will be performed using Kaplan‐Meier survival curves, evaluating time‐to‐event data for each secondary efficacy endpoint. The log‐rank test used for the primary endpoint analysis will also be applied to secondary endpoints. Treatment differences will be tested at an alpha level of 0.05, employing a sequential procedure for type 1 error correction. Covariate adjustments for time‐dependent analyses will again be performed using the Cox proportional hazards model, with the HR and 95% CI reported.

For non–time‐to‐event secondary endpoints, including changes in metabolic and clinical parameters (blood pressure, HbA1c, LDL‐C) and patient‐reported outcomes, analyses will be performed using paired t‐tests and analysis of covariance (ANCOVA) models, incorporating baseline values as covariates. Treatment differences will be evaluated at a two‐sided alpha level of 0.05, applying the same hierarchical procedure used for time‐to‐event endpoints to control type I error. All analyses will follow the ITT principle, and sensitivity analyses will be conducted to assess the impact of missing data.

For the tertiary endpoint analysis, the study will evaluate changes in the MeTaRiSc from baseline to Week 84. Each participant will receive a score of 1 for each parameter above the target; the total score will then be divided by three, producing a weighted mean ranging from 0 to 1. A paired t‐test will compare the two treatment groups. Treatment differences will be tested at an alpha level of 0.025, using a sequential type 1 error correction procedure. Covariate adjustments will be made using a *t*‐test, incorporating the randomization stratification factor. The mean values for each treatment group (intervention vs. control) will be reported with the corresponding 95% CI.

All subgroup analyses will involve log‐rank tests and the hazard ratios (with 95% CIs) for primary and secondary efficacy endpoints. The predefined subgroups include sex, age (< 65 vs. ≥ 65 years), prior cardiovascular disease, presence of cardiovascular risk factors (diabetes, hypertension, chronic kidney disease), smoking history (active vs. significant past smoking), obesity/metabolic syndrome, and prior statin use.

The present study was designed in accordance with the NIH‐FDA Clinical Trial Protocol Template. A structured protocol and a Statistical Analysis Plan (SAP) were developed prior to participant enrollment, specifying the analytic approach for all predefined endpoints, including sensitivity and subgroup analyses.

## 10 Discussion

The TAMIS trial is a prospective, randomized, open‐label, controlled clinical trial designed to evaluate the impact of a remotely delivered lipid, glucose, and hypertension management program on adult diabetic patients in Brazilian communities. The study hypothesizes that this intervention will enhance the provision of care throughout the health system, improving accessibility and democratizing health care services. Additionally, remote monitoring is expected to facilitate pharmacotherapeutic follow‐up and optimize treatment strategies.

In recent years, significant research has explored the role of technology in remote monitoring and chronic disease management [17]. Telemonitoring has emerged as an innovative approach to empower patients in self‐managing their health conditions. Ding et al. conducted a multicenter randomized controlled trial (RCTs) involving 184 participants over a 6‐month follow‐up to assess adherence to self‐management recommendations within a telemonitoring‐enhanced care program for chronic HF (CHF). The study demonstrated significant improvements in CHF self‐management behaviors, particularly in health maintenance, medication adherence, and dietary practices, reinforcing the potential of telemonitoring to support patients in chronic disease management [18].

A recent RCT conducted in Peru evaluated a home‐based telemonitoring approach for blood pressure control in primary care patients and demonstrated a significant reduction in diastolic blood pressure (−7.2 mmHg vs. −1.2 mmHg, *p* = 0.03). Despite its short 4‐week duration and small sample size, the study reinforces the feasibility of digital health interventions within LMIC health systems and underscores the importance of broader, longer, and more integrated approaches such as TAMIS [19].

Similarly, Liu et al. conducted a systematic review and meta‐analysis of RCTs investigating self‐management interventions for HF patients that incorporated communication technology. The primary outcomes assessed included all‐cause readmission rate and all‐cause mortality, while secondary outcomes encompassed quality of life, self‐care behaviors, HF knowledge, and medication adherence. Among the 1884 screened, 24 RCTs met the inclusion criteria, representing 9634 participants, with 4820 allocated to the intervention group and 4814 to usual care. The meta‐analysis revealed that cardiovascular mortality was significantly lower in the intervention group (OR 0.74, 95% CI 0.59−0.92, *p* = 0.008), as well as all‐cause readmission rates (OR 0.82, 95% CI 0.73−0.93, *p* = 0.002) [18]. These findings highlight the clinical benefits of integrating technology‐based self‐management approaches in patients with chronic conditions.

The adoption of information and communication technologies in health care can reduce or eliminate barriers to face‐to‐face consultations, improving treatment accessibility, decreasing wait times, and lowering hospitalization rates. Additionally, digital health interventions can be more cost‐effective than conventional in‐person approaches. However, despite these benefits, the successful integration of self‐management interventions into routine clinical practice remains challenging [18].

The TAMIS trial represents a pioneering effort to validate the efficacy of remote monitoring technologies and promote their adoption in low—and middle—income countries (LMICs), where access to health care facilities is often limited. Implementing these strategies in resource‐constrained settings could be pivotal for improving health outcomes and reaching vulnerable populations. This approach is particularly relevant for diseases with high morbidity and mortality, such as dyslipidemia, diabetes, and SAH.

Access as a determinant of treatment effectiveness was demonstrated by the BARBER‐1 trial, which showed an improvement in hypertension control when care was delivered in community‐based settings routinely accessed by patients. In that study, barbershops functioned as sites for monitoring and referral, reversing the conventional model in which patients are required to seek out health services [20]. Similarly, the TAMIS platform integrates clinical decision support, remote monitoring, and technology‐mediated communication into the patient's environment, increasing access, reducing friction with the system, and enabling therapeutic adjustments.

This study has inherent limitations related to its open‐label design, including the risk of ascertainment bias in both clinical and self‐reported outcomes. Additionally, potential loss to follow‐up exceeding the estimated 5% may introduce attrition bias, thereby compromising internal validity and reducing the statistical power of the analysis. These factors should be considered when interpreting the results, despite the methodological strategies implemented to mitigate their impact.

The study aims to improve patient education, facilitate collaboration between primary care providers and specialists, and promote a more flexible, convenient, and cost‐effective health care model, ultimately leading to better patient outcomes.

## 11 Conclusion

This study hypothesizes that technology‐based remote management for prevalent chronic diseases, including hypertension, diabetes mellitus, and dyslipidemia, may lead to different impacts on the incidence of significant clinical outcomes compared to usual care, potentially offering valuable insights into the role of digital health interventions.

## Author Contributions

Concept and design: Renato de Carvalho Barros, Luiz Sérgio F. de Carvalho. Acquisition of data: not applicable. Analysis or interpretation of data: not applicable. Drafting of the manuscript: Renato de Carvalho Barros, Evellyn Mariana, Yasmim Botelho, Thaiene M. M. Severino, Robson Conceição Silva, Lucila de Jesus Almeida, Mariana Guimarães Souza de Oliveira, Ana Carolina Augusto, Catarina Ferraz. Critical revision of the manuscript for important intellectual content: Luiz Sérgio F. de Carvalho, Alexandre Anderson S. M. Soares, Sérgio Henrique Rodolpho Ramalho, Ana Claudia C. Nogueira, Andrei C. Sposito, Alessandra M. Campos‐Staffico. Statistical analysis: not applicable. Provision of study materials or patients: Renato de Carvalho Barros, Luiz Sérgio F. de Carvalho, Giselle Pinto. Supervision: Luiz Sérgio F. de Carvalho. Obtaining funding: Luiz Sérgio F. de Carvalho. Administrative, technical, or logistic support: Renato de Carvalho Barros, Andrea Stephanus, José Antonio Barbosa Filho, Cristiane Koeche, Bruno Gedeon, Enzo F., Gabriela de Lima, Julia Andrade Ibiapina.

## Conflicts of Interest

The authors declare no conflicts of interest.

## Supporting information

Supporting File

## Data Availability

The data that support the findings of this study are available on request from the corresponding author. The data are not publicly available due to privacy or ethical restrictions.
