# Supplementary material for: A Remote Chronic Disease Management Program to Improve Cardiovascular and Metabolic Outcomes in a Diverse Community‐Based Diabetic Population: Study Design of the TAMIS Trial
Source: J Eval Clin Pract. 2026 Apr 29;32:e70456. doi: 10.1111/jep.70456 (PMC13127239; doi:10.1111/jep.70456)

**SUPPLEMENTARY MATERIALS**

**A Remote Chronic Disease Management Program to Improve Cardiovascular and Metabolic Outcomes in a Diverse Community-Based Diabetic Population: Study Design of the TAMIS Trial**

Renato de Carvalho Barros, MD^1,8^; Andrea Stephanus, MSc^1^; José Antonio Barbosa Filho, MD^1,2^; Evellyn Mariana^1^; Yasmim Botelho^1^; Thaiene M. M. Severino ^1,2^, MD^1^; Robson Conceição Silva, MSc^1,3^; Lucila de Jesus Almeida, MD^1^; Cristiane Koeche, MSc^1^; Bruno Gedeon, MSc^1^; Mariana Guimarães Souza de Oliveira^1^; Ana Carolina Augusto^1^; Catarina Ferraz^1^; Enzo F.^1^; Gabriela de Lima^1^; Julia Andrade Ibiapina^1^; Giselle Pinto, MD^1^; Sérgio Henrique Rodolpho Ramalho, MD, MSc, PhD^9^; Ana Claudia C. Nogueira, MD, MSc, PhD^1,2,4^; Andrei C Sposito MD, PhD ^6^; Alessandra M. Campos-Staffico, BPharm, PhD^5^; Alexandre Anderson S. M. Soares, MD, PhD^1,4^; Luiz Sérgio F. de Carvalho, MD, MSc, PhD^1,2,4,6,7,9^.

1. Data Laboratory for Research on Quality of Care and Outcomes (LaDaQCOR), Catholic University of Brasília (UCB), Brasília, Brazil.

2. Research Group on Cardiovascular Diseases of Brasília, Higher School of Health Sciences (ESCS), University of the Federal District (UnDF), Brasília, Brazil.

3. Postgraduate Program in Physical Education (PPGEF), Catholic University of Brasília (UCB), Brasília, Brazil.

4. Aramari Apo Institute, Brasília, Brazil.

5. Department of Pharmaceutical Sciences, School of Pharmacy and Health Professions, Creighton University, Omaha, USA.

6. Department of Cardiology, State University of Campinas (Unicamp), Campinas (SP), Brazil.

7. Clarity Healthcare Intelligence, Campinas (SP), Brazil.

8. Post-graduation program in Medical Sciences, University of Brasília, Brasília, Distrito Federal, Brazil

9. Hospital Sírio Libanês, Brasília, Distrito Federal, Brazil

|  | Randomization  Visit (RV) | V1  W1 | V2  W6 | V3  W12 | V4  W24 | V5  W36 | V6  W56 | V7  W60 | V8  W72 | V9  W78 | V10  W84 |
| --- | --- | --- | --- | --- | --- | --- | --- | --- | --- | --- | --- |
| Informed consent | X |  |  |  |  |  |  |  |  |  |  |
| Demographic data | X |  |  |  |  |  |  |  |  |  |  |
| History of present illness | X | X | X | X | X | X | X | X | X | X | X |
| Monitor signs and symptoms | X | X | X | X | X | X | X | X | X | X | X |
| Blood sample collections | X |  |  | X | X | X | X |  |  |  | X |
| Administration of protocol therapy according to randomization |  | X | X | X | X | X | X | X | X | X | X |
| Surveillance for AE or SAE |  | X | X | X | X | X | X | X | X | X | X |
| Recording of data in database | X | X | X | X | X | X | X | X | X | X | X |
| Appraisal of study endpoints |  |  | X | X | X | X | X | X | X | X | X |
| Laboratory and clinical exams | | | | | | | | | | | |
| Complete blood count with differential cell count | X |  |  |  |  |  | X |  |  |  | X |
| Glucose | X |  |  |  |  |  | X |  |  |  | X |
| HbA1c | X |  |  | X | X | X | X |  |  |  | X |
| Lipid profile | X |  |  | X | X | X | X |  |  |  | X |
| Creatinine | X |  |  |  |  |  | X |  |  |  | X |
| Albumin/Creatinine ratio | X |  |  |  |  |  | X |  |  |  | X |
| Quality of life evaluation (EQ-5D) | X |  |  |  |  |  | X |  |  |  | X |

**Supplementary Material 1.** Schedule of Activities. Description of study procedures and measurements.

**Supplementary Material 2.** TAMIS-IA Platform

**1. Platform description**

TAMIS-IA is a web-based digital health platform developed to support remote clinical management of patients enrolled in the TAMIS Trial. The system enables bidirectional interaction between healthcare teams and patients, supporting clinical data input, remote treatment adjustment, communication, and decision support based on standardized algorithms.

**2. User interfaces**

Patients access TAMIS-IA through a secure individual login, where they are prompted to report clinical parameters such as blood pressure, glucose levels, symptoms, and medication adherence. The platform provides educational feedback and real-time alerts for critical values. Healthcare professionals and study researchers, in turn, use a dedicated dashboard to access de-identified patient data, including longitudinal trends in key metrics (e.g., SBP, DBP, LDL-C, HbA1c). The system enables remote management of therapy based on clinical algorithms and offers tools for monitoring adherence and tracking clinical events.

**3. Data flow and integration**

Clinical data entered by patients and healthcare providers are automatically uploaded to a central server and made available for review by the clinical team. Algorithms embedded in the platform generate treatment recommendations based on national and international clinical guidelines. Researchers can export data from the system in compliance with Brazilian LGPD and GCP standards.

**4. Security and compliance**

The platform is hosted on secure servers and includes user-level access control and audit trails. All stored data comply with the Brazilian General Data Protection Law (LGPD). Access is limited to authorized personnel.

**5. Features summary**

| FEATURE | DESCRIPTION |
| --- | --- |
| Data entry | Blood pressure, symptoms, glucose, medication |
| Alerts and flags | Real-time alerts for out-of-range values |
| Remote management | Clinicians can adjust therapy using decision trees |
| Dashboard analytics | Trend graphs, MeTaRiSc tracking, adherence scores |
| Communication | Messaging between patient and monitor |
| Export | Secure CSV/TAMIS-AI-compatible data export |

**Supplementary Material 3.** Safety Assessments

**1. Specification of variables and procedures**

Safety assessments will include adverse events, clinical laboratory measurements (chemistry, hematology) and vital signs (systolic and diastolic blood pressure). A complete medical, surgical, and family history will be completed at the randomization visit. All laboratory test results must be evaluated by the investigator as to their clinical significance. Any observations at physical examinations or laboratory values considered by the investigator to be clinically significant should be considered an adverse event.

**2. Definition of adverse events**

An adverse event is defined as any untoward medical occurrence characterized by unexpected events that takes place during the treatment with the medication under investigation. It does not necessarily have a causal relationship with the medication. An adverse event can be any unintended and/or unfavorable symptom, sign (including an abnormal laboratory finding), deterioration of underlying conditions, or disease temporarily associated with the use of an investigational medication product. All adverse events are to be recorded by investigators on the appropriate CRF during each of the visits. Each adverse event will be evaluated for duration, intensity, and causal relationship with the study medication or other factors. The investigators will refrain from adding signs and symptoms as separate individual adverse events if they can be unified under a single syndrome or diagnosis. If that is not possible, each will be filed as a separate adverse event. The investigator will classify each event as either mild, moderate, or severe (as specified in Section 2.1 below) and categorize each adverse event as to its potential relationship to study medication (i.e., yes or no).

**2.1. Classification of an adverse event**

**2.1.1. Severity of event**

The adverse events will be graded according to the following guidelines to describe severity.

- Mild – Events require minimal or no treatment and do not interfere with the participant’s daily activities.
- Moderate – Events result in a low level of inconvenience or concern with the therapeutic measures. Moderate events may cause some interference with functioning.
- Severe – Events interrupt a participant’s usual daily activity and may require systemic drug therapy or other treatment. Severe events are usually potentially life-threatening or incapacitating. Of note, the term “severe” does not necessarily equate to “serious”.

**2.1.2. Causality assessment**

The relationship of an adverse events to the study intervention will be assessed by the clinician who examines and evaluates the participant based on temporal relationship and his/her clinical judgment. This relationship is to be assessed according to the following definitions:

- Related – There is a temporal relationship between the study drug administration and the adverse event is consistent with a causal relationship and no other cause is identified (concomitant drugs, therapies, complications, etc.).
- Not Related – The temporal relationship between the study drug administration and the adverse event is not consistent with a causal relationship and another cause is suspected (concomitant drugs, therapies, complications, etc.).

The following factors should also be considered:

- The temporal sequence from administration of study medication
- The adverse event should occur after the study medication is given and the time course between study medication exposure and to event should be assessed in the clinical context of the event
- Each report should be evaluated in the context of the natural history of the disease being treated
- Any underlying, concomitant, or intercurrent diseases
- All concomitant medications that compose the participant’s therapeutic regimen or underlying diseases should be evaluated for the possibility that they may be a recognized cause of the event in question
- The pharmacology and pharmacokinetics of the study medication should be considered
- The question of a finitude of resources for therapeutic management will also be evaluated as possible explanations for event in question. For example, there may be times in which scarcity of clinical resources may be a driver for events in the right clinical context of this pandemic.

**2.1.3. Unexpected adverse events**

An adverse event will be considered unexpected if the nature, severity, or frequency of the event is not consistent with the risk information previously described for the study intervention.

**3. Definition of serious adverse events**

A serious adverse event is, in the view of either the investigator or sponsor, any event that results in any of the following outcomes: death, a life-threatening adverse event (i.e., if any worse it would have resulted in death), re-hospitalization or prolongation of existing hospitalization, a persistent or significant incapacity or substantial disruption of the ability to conduct normal life functions, or a congenital anomaly/birth defect. Important medical events that may not result in death, be life-threatening, or require hospitalization may be considered serious when, based upon appropriate medical judgment, they may jeopardize the participant and may require medical or surgical intervention to prevent one of the outcomes listed in this definition.

**4. Safety endpoints**

- Cumulative incidence of adverse events;
- Cumulative incidence of serious adverse events.

**5. Adverse event reporting**

**5.1. Time period and frequency for event assessment and follow-up**

The occurrence of all adverse events may come to the attention of the study personnel during visits and interviews of a study participant or upon review by a study monitor. Adverse events that occur from the time of informed consent and until 7 days after the administration of study interventions. Serious adverse events that occur from the time of informed consent and until 30 days after the administration of study interventions will be reported to the Sponsor within 24 hours of the knowledge of the occurrence. Study endpoints that are serious adverse must be reported in accordance with the protocol unless there is evidence suggesting a causal relationship between the study intervention and the event (e.g., death from anaphylaxis). In that case, the investigator must immediately report the event to the Sponsor.

Events will be followed for outcome information until resolution or stabilization. All serious adverse events will be followed until satisfactory resolution, stabilization, or until the site investigator deems the event to be chronic. Other supporting documentation of the event may be requested by the Coordinating Center and should be provided as soon as possible.

**5.2. Adverse event reporting**

All adverse events that do not meet criteria for a serious adverse event will be captured on the appropriate case report form (CRF) by study personnel. This report will include event description, time of onset, clinician’s assessment of severity, relationship to study product, and time of resolution of the event. All adverse events will be documented while on study regardless of causality.

**5.3 Serious adverse event reporting**

The study investigator is mandated to submit an Unanticipated Adverse Device Effect form to the study Sponsor and to submit a serious adverse event reports to the Institutional Review Board (IRB) or Independent Ethics Committee (IEC) in accordance with local requirements. Cases will be unblinded for reporting purposes as required. The study sponsor will be responsible for notifying National Sanitary Surveillance Agency (ANVISA) of any unexpected fatal or life-threatening suspected adverse reaction as soon as possible, but in no case later than 7 calendar days after the sponsor’s initial receipt of the information.

**Supplementary Material 4.** Sample size estimation: impact of hazard ratios for death and hospitalization on power calculation.


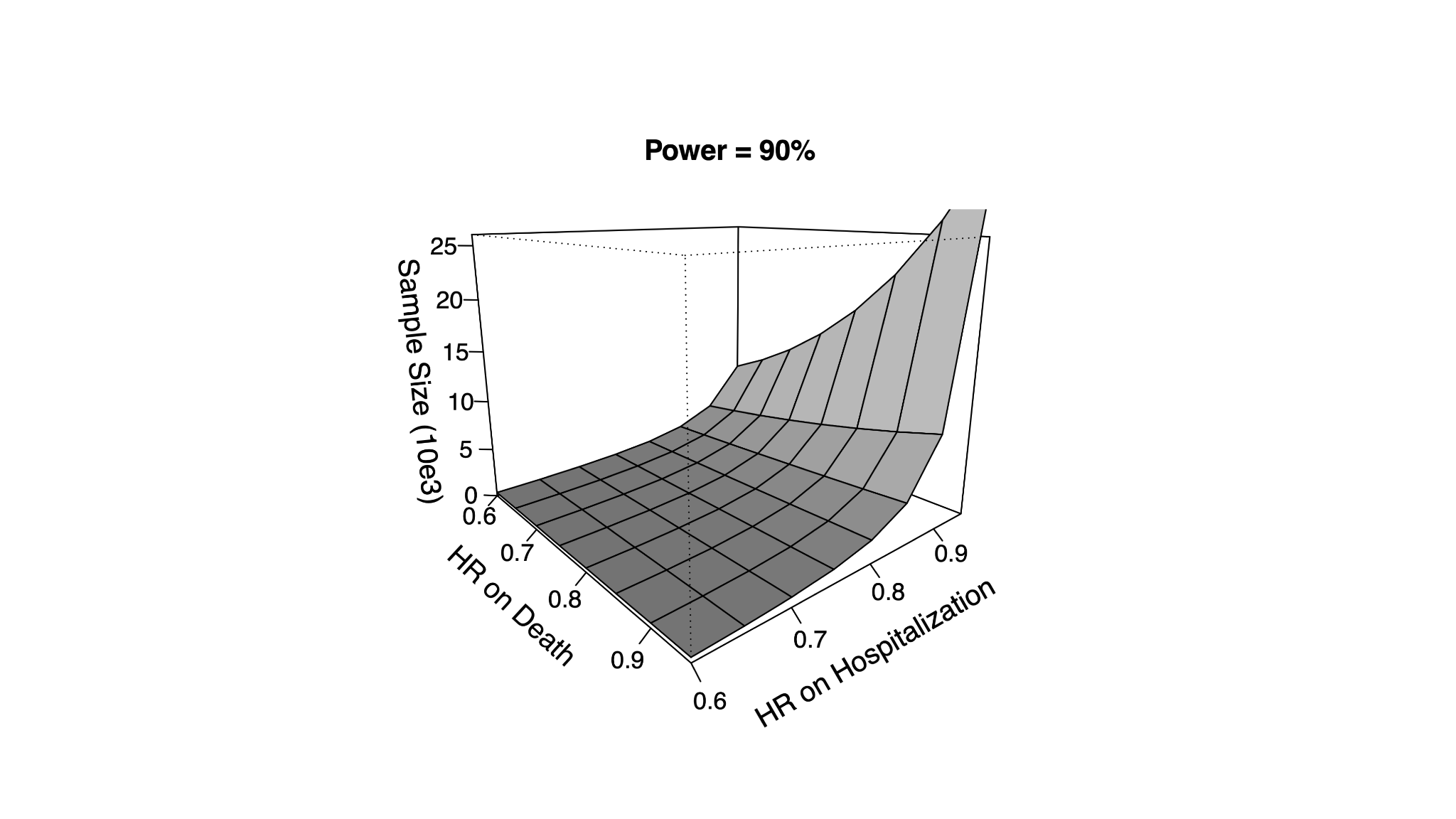

Supplement: Supplementary file 1 — Supporting File [file JEP-32-0-s001.docx]
